# Copy Number Variation and Clinical Outcomes in Patients With Germline *PTEN* Mutations

**DOI:** 10.1001/jamanetworkopen.2019.20415

**Published:** 2020-01-31

**Authors:** Lamis Yehia, Marilyn Seyfi, Lisa-Marie Niestroj, Roshan Padmanabhan, Ying Ni, Thomas W. Frazier, Dennis Lal, Charis Eng

**Affiliations:** 1 Genomic Medicine Institute, Lerner Research Institute, Cleveland Clinic, Cleveland, Ohio; 2 Cologne Center for Genomics, University of Cologne, Cologne, Germany; 3 Department of Quantitative Health Sciences, Lerner Research Institute, Cleveland Clinic, Cleveland, Ohio; 4 Autism Speaks, Cleveland, Ohio; 5 Department of Psychology, John Carroll University, University Heights, Ohio; 6 Epilepsy Center, Neurological Institute, Cleveland Clinic, Cleveland, Ohio; 7 Stanley Center for Psychiatric Research, Broad Institute of MIT (Massachusetts Institute of Technology) and Harvard, Cambridge, Massachusetts; 8 Taussig Cancer Institute, Cleveland Clinic, Cleveland, Ohio; 9 Department of Genetics and Genome Sciences, Case Western Reserve University School of Medicine, Cleveland, Ohio; 10 Germline High Risk Cancer Focus Group, Case Comprehensive Cancer Center, Case Western Reserve University, Cleveland, Ohio

## Abstract

**Importance:**

*PTEN* is among the most common autism spectrum disorder (ASD)–predisposition genes. Germline *PTEN* mutation carriers can develop malignant neoplasms and/or neurodevelopmental disorders such as ASD and developmental delay. Why a single gene contributes to disparate clinical outcomes, even in patients with identical *PTEN* mutations, remains unclear.

**Objective:**

To investigate the association of copy number variations (CNVs), altered numbers of copies of DNA sequences within the genome, with specific phenotypes in patients with germline *PTEN* mutations.

**Design, Setting, and Participants:**

This prospective cohort study examined genome-wide microarrays performed on blood-derived DNA to detect germline CNVs from September 1, 2005, through January 3, 2018. Multicenter accrual occurred from community and academic medical centers throughout North America, South America, Europe, Australia, and Asia. Participants included patients with *PTEN* hamartoma tumor syndrome (PHTS) (n = 481), molecularly defined as carrying germline pathogenic *PTEN* mutations. Data were analyzed from November 14, 2018, to August 1, 2019.

**Exposures:**

Detection of CNVs from patient-derived germline DNA.

**Main Outcomes and Measures:**

Prevalence of pathogenic and/or likely pathogenic CNVs in patients with PHTS and association with ASD/developmental delay and/or cancer, ascertained through medical records and pathology reports.

**Results:**

The study included 481 patients with PHTS (mean [SD] age, 33.2 [21.6] years; 268 female [55.7%]). The analytic series consisted of 309 patients with PHTS and genetically determined European ancestry. Patients were divided into 3 phenotypic groups, excluding family members within each group. These include 110 patients with ASD/developmental delay, 194 without ASD/developmental delay, and 121 with cancer (of whom 116 were in the no ASD/developmental delay group). Genome-wide evaluation of autosomal CNVs indicated an increased CNV burden, particularly duplications in genic regions, in patients with ASD/developmental delay compared with those without ASD/developmental delay (odds ratio [OR], 1.9; 95% CI, 1.1-3.4; *P* = .03) and those with cancer (OR, 2.5; 95% CI, 1.3-4.6; *P* = .003). Eleven of the 110 patients (10.0%) with ASD/developmental delay carried pathogenic and/or likely pathogenic CNVs associated with neurodevelopmental disorders, compared with 5 of 194 (2.6%) without ASD/developmental delay (OR, 4.2; 95% CI, 1.4-13.7; *P* = .008) and 2 of 121 (1.7%) with cancer (OR, 6.6; 95% CI, 1.6-44.5; *P* = .007). Evidence of an association between pathogenic and/or likely pathogenic CNVs and PHTS with ASD/developmental delay was further supported in a validation series of 69 patients with PHTS of genetically determined non-European ancestry.

**Conclusions and Relevance:**

These findings suggest that copy number variations are associated with the ASD/developmental delay clinical phenotype in PHTS, providing proof of principle for similarly heterogeneous disorders lacking outcome-specific associations.

## Key Points

**Question:**

Are copy number variations associated with specific clinical outcomes in patients with germline *PTEN* mutations?

**Findings:**

In this cohort study of 481 patients with germline *PTEN* mutations, pathogenic and/or likely pathogenic copy number variations associated with neurodevelopmental disorders were found in 10.0% of patients with autism spectrum disorder and/or developmental delay. Pathogenic and/or likely pathogenic copy number variations were found in 2.6% of patients without autism spectrum disorder and/or developmental delay and 1.7% of patients with cancer.

**Meaning:**

These findings suggest that copy number variations are associated with the autism spectrum disorder and/or developmental delay phenotype in patients with germline *PTEN* mutations.

## Teaser

This cohort study assesses whether copy number variations are associated with specific phenotypes in patients with germline *PTEN* mutations.

## Introduction

The triumph of using genetics in precision medicine is the ability to stratify individuals at very high risk of disease and thereby enact specific gene-informed medical management.^1,2^ In the case of heritable disease, genetics also allow testing of family members to determine with 100% accuracy whether they are at risk of disease. However, for any single individual carrying a mutation, we can estimate risk by probabilities but not with certainty. For example, a germline mutation in gene X may give an 80% lifetime risk of disease Y, but for the single patient with that germline mutation, the risk of developing disease Y will be 100% or 0%. Searching for genomic modifiers of heritable disease risk at the individual level in humans has proven challenging.^1,3^ This makes estimation of clinical outcomes difficult, especially for phenotypically variable disorders. As such, why a mutation in 1 particular gene predisposes to disparate clinical outcomes, including in patients with identical mutations, remains poorly understood.

*PTEN* germline mutations are among the most common causes of autism spectrum disorder (ASD), accounting for approximately 2% to 5% of cases,^4,5^ yet originally identified in a subset of relatively rare syndromes predisposing to breast, thyroid, and other cancers.^6^ The *PTEN*-related ASD-cancer phenotypic dichotomy and inability to preemptively estimate disease outcomes pose a challenge for more timely and precise clinical management. The concept of *PTEN* hamartoma tumor syndrome (PHTS [OMIM 158350]) was proposed to encompass any clinical disorder with germline *PTEN* mutation on molecular genetic testing, regardless of phenotype.^7,8^ Based on this broad clinical spectrum and unified genetic etiology, PHTS serves as a useful disease model to identify modifiers of variable heritable disease risk at the individual level.

Copy number variation (CNV), a common type of structural variation in the human genome, is considered an important contributor to nonsyndromic idiopathic ASD and sporadic cancer.^9,10,11^ Copy number variations have also been associated with complex disorders, particularly those involving developmental delay, intellectual disabilities, and/or congenital anomalies.^12,13,14^ As such, the purpose of this study was to address the hypothesis that specific CNV associations in patients carrying germline *PTEN* mutations are associated with development of specific clinical phenotypes at an individual level. Thus, we performed a genome-wide investigation of whether pathogenic and/or likely pathogenic CNVs may be associated with particular clinical outcomes in patients with germline *PTEN* mutations.

## Methods

### Patients

A total of 6782 patients were prospectively accrued from September 1, 2005, through January 3, 2018, and provided informed written consent to participate. This prospective cohort study was approved by the Cleveland Clinic institutional review board. This study followed the Strengthening the Reporting of Observational Studies in Epidemiology (STROBE) reporting guideline for cohort studies.

 Inclusion criteria for enrollment included meeting at least the relaxed International Cowden Consortium operational diagnostic criteria, meaning full diagnostic criteria minus 1 feature, termed *Cowdenlike syndrome*^15^; having macrocephaly plus neurodevelopmental disorders (eg, ASD, developmental delay [DD], mental retardation) and/or penile freckling; or the presence of a known pathogenic germline *PTEN* mutation (patients referred to the *PTEN* Multidisciplinary Clinic at the Cleveland Clinic).^16,17^ These patients were broadly recruited from community and academic medical centers throughout North America, South America, Europe, Australia, and Asia, as noted by Tan et al.^16,17^ After informed consent was obtained, checklists to document the presence or absence of specific features were completed by specialist genetic counselors or physicians concurrently with withdrawal of blood specimen (team led by C.E., medical director, PTEN/Cowden Multidisciplinary Clinic, Center for Personalized Genetic Healthcare, Genomic Medicine Institute, Cleveland Clinic, Cleveland, Ohio). Specialist genetics staff reviewed all checklists and corresponded with the enrolling center if necessary to obtain further primary documentation of medical records and, for cancer, pathology reports, for phenotype confirmation with patient consent.^16^ For each consenting patient, we reviewed medical records, including pedigrees, clinical genetic testing reports, and clinical notes associated with genetics evaluations, ASD *Diagnostic and Statistical Manual of Mental Disorders* (Fourth Edition, Text Revision) criteria, and/or genetic counseling visits, where applicable. We followed up this cohort prospectively for development of phenotypic features.

### *PTEN* Mutation and Deletion Analysis

All eligible patients underwent germline *PTEN* (NM_000314.8) mutation and deletion analysis. Genomic DNA was extracted from peripheral blood samples using standard protocols. Mutation analysis was standardly performed, with a combination of denaturing gradient gel electrophoresis, high-resolution melting curve analysis (Idaho Technology), and direct Sanger sequencing (ABI 3730xl; Applied Biosystems, Life Technologies).^18^ Deletion analysis using the multiplex ligation-dependent probe amplification assay was performed with the multiplex ligation-dependent probe amplification kit (P158; MRC-Holland) according to manufacturer protocol.^18^ All patients underwent polymerase chain reaction–based Sanger sequencing of the *PTEN* promoter region as previously described.^18^ Only cases with pathogenic or likely pathogenic germline *PTEN* mutations were included in the analytic sample. To be conservative, individuals with *PTEN* promoter variants were considered mutation positive only if the underlying variants had been previously associated with PHTS or known to affect *PTEN* function.^16,17,19,20^ Pathogenicity predictions were ascertained by reports from orthogonal testing in a Clinical Laboratory Improvement Amendments of 1988–certified facility, ClinVar database classifications, and/or the ClinGen gene-specific criteria for *PTEN* variant curation.^21^ The resultant data set represents, to our knowledge, the largest deeply phenotyped worldwide series of patients with PHTS from the International Cowden Consortium.^15^

### Genotyping, CNV Calling, and Quality Control

We evaluated DNA quality (A260/A280) using spectrophotometry (NanoDrop 1000; Thermo Fisher Scientific) and quantity using a double-stranded DNA high-sensitivity assay kit (Qubit; Thermo Fisher Scientific). DNA samples were all genotyped on a commercially available genotyping array (Infinium Global Screening Array-24, version 1.0; Illumina) at the Broad Institute Genomic Services, Cambridge, Massachusetts. We used a total of 642 824 markers for quality control. To correct for population stratification, single-nucleotide polymorphism (SNP) genotype quality control was performed. We excluded samples with a call rate of less than 0.91 or with discordant sex. We focused on autosomal CNVs owing to higher fidelity of CNV calling compared with sex chromosomes.^22^ To perform principal component analysis to assess population stratification, we filtered autosomal SNPs with low genotyping rates (>0.98) and deviation from Hardy-Weinberg equilibrium (*P* ≤ .001) before pruning SNPs for linkage disequilibrium using default parameters in PLINK, version 1.927 (--indep-pairwise 200 100 0.2).^23^ We used the PennCNV algorithm^24^ to generate GC DNA nucleotide wave-adjusted log R ratio intensity files and to call CNVs in all samples. A custom population B-allele frequency file was generated before CNV calling. We merged adjacent CNV calls if the number of intervening markers was less than 20% of the total number than when both segments were combined. Next, we performed intensity-based quality control to exclude samples with low quality based on waviness factor, log R ratio SD, and B-allele frequency drift. These thresholds were calculated by taking the median plus 3 SD to identify outliers.^25^ After intensity-based quality control, all samples had a log R ratio SD of less than 0.25, an absolute waviness factor of less than 0.04, and a B-allele frequency drift of less than 0.007. We excluded samples with an excessively high CNV load (>100) as determined empirically by visual inspection of CNV distribution across a combined set of samples. None of the PHTS samples had a CNV load above this threshold (with a maximum of 16 CNVs detected in 1 patient). Finally, we excluded CNVs spanning less than 20 markers, CNVs of less than 20 Kb in length, CNVs with SNP density (number of markers/CNV length) of less than 0.0001, and/or CNVs with greater than 50% of total length overlapping artifactual regions in SNP-based CNV calling.^26,27^ The final data set consisted of 309 patients of genotype-determined European ancestry with at least 1 CNV call (eFigure 1 in the Supplement).

### Genome-wide CNV Burden Analysis

We measured CNV burden among patients with PHTS and ASD and/or developmental delay (ASD/DD group), those without ASD or developmental delay (no ASD/DD group), and those with cancer. Genome-wide burden was also analyzed for only rare CNVs (excluding CNVs overlapping >80% of regions known to be recurrent in the general population^28^). Duplications and deletions were analyzed in combination as well as separately. Moreover, within each category (duplications and deletions), genic and nongenic CNVs were analyzed in combination as well as separately. For all comparisons, we counted the number of unrelated probands with such genomic events within each phenotype group.

### CNV Annotation and Identification of Clinically Relevant CNVs

A CNV was considered as genic if it overlapped more than 80% of a gene^29^; otherwise it was annotated as nongenic. Cancer-relevant loci included 46 genes associated with Cowden syndrome component cancers,^17^ of which 24 are clinically actionable cancer-related genes according to the American College of Medical Genetics and Genomics guidelines.^30,31^ Genes associated with ASD were curated from the Simons Foundation Autism Research Initiative Gene module.^32^ Only syndromic (category S), high-confidence (category 1), and strong candidate (category 2) genes were retained. We also interrogated regions associated with genomic disorders, congenital malformations, and neurodevelopmental phenotypes, including 48 loci from DECIPHER (Database of Chromosomal Imbalance and Phenotype Using Ensembl Resources), 298 genes from the Developmental Disorders Genotype-Phenotype Database, and 92 loci of pathogenic CNVs from the UK Biobank.^33,34,35,36,37^ Pathogenicity and likely pathogenicity of previously unreported CNVs were estimated based on size and gene content according to the American College of Medical Genetics and Genomics guidelines.^34^ We used Human Phenotype Ontology terms for standard annotation of CNVs with associated phenotypic abnormalities.^38^

### Statistical Analysis

Data were analyzed from November 14, 2018, to August 1, 2019. Patient demographic characteristics were reported by age, sex, and clinical phenotypes. OpenEpi software was used to calculate odds ratios (ORs) for burden and enrichment analyses. For analyses among different patient phenotype groups, 2 × 2 tables were used to calculate ORs. The 95% CIs and corresponding *P* values were calculated using the mid-*P* exact test. The nonparametric Mann-Whitney test was used to analyze CNV burden per individual among the different patient phenotype groups. All *P* values were 2-sided and considered to be significant at *P* < .05. We used Prism 8, version 8.1.1 (GraphPad) to generate box-and-whisker and forest plots.

## Results

### Patient Characteristics and *PTEN* Mutation Spectrum

At baseline, we prospectively accrued 481 patients with PHTS (mean [SD] age at consent, 33.2 [21.6] years; 268 female [55.7%] and 213 male [44.3%]) (eTable 1 in the Supplement). The Cleveland Clinic score, a quantitative surrogate of age-related PHTS disease burden,^16^ ranged from 0 to 69 (mean [SD], 20 [13]), corroborating the broad phenotypic spectrum, including participants with unexpectedly mild disease manifestations (eg, absence of early-onset component cancers and neurotypical development) despite their underlying pathogenic germline *PTEN* mutations. Patients with PHTS were stratified into 3 phenotypically ascertained groups, including the ASD/DD group, no ASD/DD group, and cancer group (a subset of the no ASD/DD group). We did not include a no cancer group owing to the inability to ascertain cancer diagnoses, particularly in pediatric, adolescent, and young adult patients. Importantly, the *PTEN* mutation spectra were similar across the 3 PHTS phenotype groups (eFigure 2 in the Supplement).

### Genome-wide CNV Burden in Patients With PHTS

**Figure 1.**
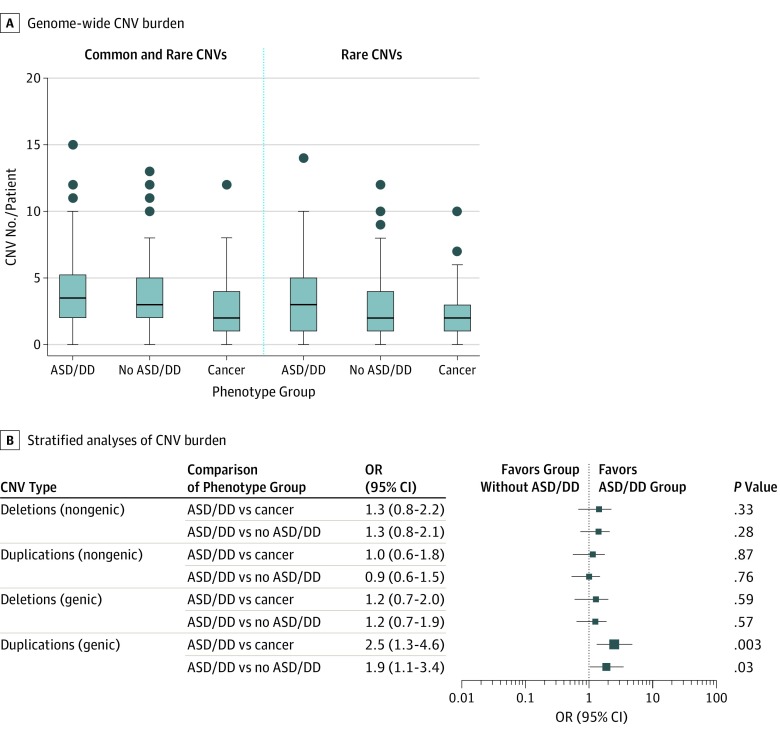
Genome-wide Copy Number Variation (CNV) Landscape Showing Phenotype-Specific Associations in *PTEN* Hamartoma Tumor Syndrome (PHTS) A, Genome-wide CNV burden representing the total number of CNVs in each patient within the 3 PHTS phenotype groups. The line in the middle of each box is plotted at the median. Each box extends from the 25th to 75th percentiles (interquartile range [IQR]). Whiskers and outliers (circles) are plotted according to the Tukey method. The upper whiskers extend to the largest value no further than 1.5 times the IQR. The lower whiskers extend to the smallest value no further than 1.5 times the IQR. B, Stratified analyses showing CNV burden (duplications and deletions) in genic and nongenic genomic regions, accounted for by the number of unrelated patients carrying such CNVs within each clinical phenotype group. The size of each box is proportional to the respective odds ratio (OR) depicted in the adjacent table. ASD indicates autism spectrum disorder; DD, developmental delay.

The analytic sample consisted of 309 patients with PHTS (Table 1) of genetically determined European ancestry (mean [SD] age at consent, 32.0 [21.0] years; 167 female [54.0%] and 142 male [46.0%]). Phenotypically ascertained groups consisted of 110 unrelated patients in the ASD/DD phenotype group, 194 unrelated patients in the no ASD/DD phenotype group, and a subset of 121 unrelated patients, including 116 in the no ASD/DD group, with cancer. To determine the genome-wide CNV burden, we quantified the total number of CNVs in each participant within the 3 PHTS phenotype groups (Figure 1A). Patients in the ASD/DD group had an overall increased CNV burden per individual (median, 3.5; range, 0-15) compared with those in the no ASD/DD group (median, 3.0; range, 0-13; Mann-Whitney 2-sided test comparing ranks, *P* = .04) and the subset with cancer (median, 2.0; range, 0-12; Mann-Whitney 2-sided test, *P* = .002). Similarly, patients in the ASD/DD group had an overall increased burden of rare CNVs per individual (median, 3.0; range, 0-14) compared with those in the no ASD/DD group (median, 2.0; range, 0-12; Mann-Whitney 2-sided test comparing ranks, *P* = .01) and the subset with cancer (median, 2.0; range, 0-10; Mann-Whitney 2-sided test, *P* < .001). No difference was observed in CNV burden among patients in the no ASD/DD group (median, 3.0; range, 0-13) compared with the subset with cancer (median, 2.0; range, 0-12; Mann-Whitney 2-sided test, *P* = .15). Similarly, no difference was observed in rare CNV burden among patients in the no ASD/DD group (median, 2.0; range, 0-12) compared with the subset with cancer (median, 2.0; range, 0-10; Mann-Whitney 2-sided test, *P* = .19). Stratified analyses according to CNV type and genomic locus revealed that patients in the ASD/DD group had a significant enrichment of duplications in genic regions (Figure 1B), compared with patients in the no ASD/DD group (OR, 1.9; 95% CI, 1.1-3.4; *P* = .03) and the subset with cancer (OR, 2.5; 95% CI, 1.3-4.6; *P* = .003).

**Table 1.** Clinical Characteristics of the Analytic Series of 309 Patients With PHTS and European Ancestry

Clinical Phenotypic Characteristics	Data
**Sex, No. (%)**	
** Female**	167 (54.0)
** Male**	142 (46.0)
**Age at consent, mean (SD) [range], y**	32 (21) [1-85]
**No. of germline *PTEN* mutations**	
** Promoter**	8
** Missense**	99
** Nonsense**	86
** Splice site**	28
** Frameshift truncating**	63
** Insertions and deletions**	25
**Neurodevelopmental features, No. (%)**	110 (35.6)
** Autism spectrum disorder**	45 (14.6)
** Global developmental delay**	56 (18.1)
** Variable delay**	25 (8.1)
** Mental retardation**	9 (2.9)
** Learning disabilities**	9 (2.9)
**Cancer, No. (%)**^**a**^	130 (42.1)
**PHTS component malignant neoplasms, No. (%)**	
** Breast cancer**	58 (18.8)
** Thyroid cancer**	36 (11.7)
** Renal cell cancer**	25 (8.1)
** Endometrial cancer**	27 (8.7)
** Colon cancer**	10 (3.2)
** Melanoma**	10 (3.2)
**Nonmalignant features, No. (%)**^**b**^	78 (25.2)
** Macrocephaly**	70 (22.7)
** Dermatologic features**^**c**^	42 (13.6)
** Arteriovenous malformations**	10 (3.2)
** Hemangiomas**	13 (4.2)
** Polyposis**	38 (12.3)
** Benign breast features**^**d**^	21 (6.8)
** Benign thyroid features**^**e**^	49 (15.9)
** Lhermitte-Duclos disease**	9 (2.9)

Abbreviation: PHTS, *PTEN* hamartoma tumor syndrome.

^a^ Includes 9 patients with neurodevelopmental disorders who have also been diagnosed with cancer.

^b^ Includes patients without a personal history of neurodevelopmental disorders or cancer at the time of the last clinical visit and/or follow-up.

^c^ Includes trichilemmoma, acral keratosis, papillomatous papules, and genital lentiginosis (penile freckling in males).

^d^ Includes breast fibroadenoma, fibrocystic breast disease, breast papilloma, breast hamartoma, atypical ductal hyperplasia, and typical ductal hyperplasia.

^e^ Includes thyroid nodules, goiter, and Hashimoto thyroiditis.

### Clinically Relevant CNVs in Patients With PHTS

We identified 3 of 309 patients with PHTS (1.07%) and pathogenic and/or likely pathogenic CNVs affecting genes associated with *PTEN-*related cancers^17^ or other inherited cancer syndromes according to the American College of Medical Genetics and Genomics guidelines.^30,31^ These included 2 *BMPR1A* (OMIM 601299) deletions and 1 *BRCA1* (OMIM 113705) deletion. We could not ascertain cancer diagnoses because all 3 CNV carriers were children or young adults (aged 3, 6, and 21 years) without a personal history of cancer at the time of consent (eTable 2 in the Supplement). The 2 patients with PHTS and *BMPR1A* deletions upstream of their individual *PTEN* deletion had juvenile polyposis, as is often observed in chromosome 10q23 microdeletions involving both genes (OMIM 612242). Notably, no pathogenic and/or likely pathogenic CNVs involving known cancer-associated genes were identified among patients in the subset with cancer.

**Figure 2.**
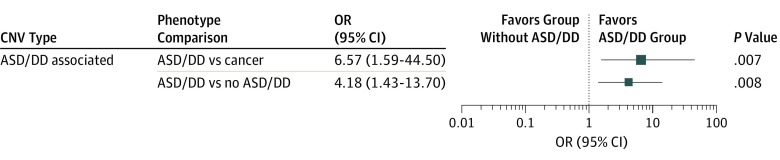
Enrichment of Clinically Relevant Copy Number Variations (CNVs) in Patients With *PTEN* Hamartoma Tumor Syndrome (PHTS) and Autism Spectrum Disorder/Developmental Delay (ASD/DD) Pathogenic and/or likely pathogenic CNVs associated with neurodevelopmental disorders were found in 11 of 110 patients in the ASD/DD group (10.0%), 5 of 194 patients in the no ASD/DD group (2.6%), and 2 of 121 patients in the subgroup with cancer (1.7%). The size of each box is proportional to the respective odds ratio (OR) depicted in the adjacent table.

We then investigated pathogenic and/or likely pathogenic CNVs involving genes implicated in the etiology of idiopathic nonsyndromic and syndromic ASD/developmental delay and/or neurodevelopmental disorders as well as known pathogenic CNVs associated with ASD, developmental delay, and neurodevelopmental disorders. We found 11 of 110 patients in the ASD/DD group (10.0%) harbored such pathogenic and/or likely pathogenic CNVs, compared with 5 of 194 patients in the no ASD/DD group (2.6%) (OR, 4.2; 95% CI, 1.4-13.7; *P* = .008) and 2 of 121 (1.7%) patients in the subset with cancer (OR, 6.6; 95% CI, 1.6-44.5; *P* = .007) (Figure 2). The CNV carriers included 10 male and 6 female patients with PHTS (mean [SD] age at consent, 22 [22.1] years). The CNVs included genomic loci associated with other genomic syndromes with overlapping clinical features, such as chromosome 8p23.1 duplication syndrome and chromosome 1q21.1 thrombocytopenia-absent radius syndrome (Table 2 and Table 3).

**Table 2.** Pathogenic and Likely Pathogenic CNVs Associated With Neurodevelopmental Disorders

Patient Identification	Phenotype Group	CNV (Size)	Genomic Coordinates (hg19)	Associated Genes	SFARI Gene Score	Associated Source or Genomic Syndromes	Related Human Phenotype Ontology
CCF00105	ASD/DD	4p16.1 dup (3.3 Mb)	chr4:5064533-8381168	*JAKMIP1*	NA	Smith-Magenis syndrome-like (OMIM 611195; ClinVar VCV000254206.1)	HP:0001249, intellectual disability; HP:0001263, global DD
CCF00301	ASD/DD	22q11.21 del (133 Kb)	chr22:18875445-19008424	*PRODH*	Strong candidate, syndromic	PMID: 29895855	HP:0001249, intellectual disability; HP:0000729, autistic behavior; HP:0001263, global DD
CCF00547	ASD/DD	10q23.2 del (4.8 Mb)	chr10:85624500-90412968	*GRID1*	Strong candidate	10q23.2 Deletion syndrome	HP:0000256, macrocephaly; HP:0000717, autism; HP:0001263, global DD; HP:0001249, intellectual disability; HP:0200008, intestinal polyposis
		6p12.1 dup (354 Kb)	chr6:56566691-56920804	*KIAA1586*	Strong candidate	NA	NA
CCF03028	ASD/DD	10q23.2 del (3.3 Mb)	chr10:87976544-91250370	*GRID1*	Strong candidate	10q23.2-q23.31 Deletion syndrome	HP:0000256, macrocephaly; HP:0000717, autism; HP:0001263, global DD; HP:0001249, intellectual disability; HP:0200008, intestinal polyposis
CCF05681	ASD/DD	15q13.3 dup (490 Kb)	chr15:32025034-32515100	*CHRNA7*	Strong candidate	15q13.3 Duplication syndrome	HP:0003829, incomplete penetrance; HP:0000256, macrocephaly; HP:0000717, autism; HP:0001263, global DD
CCF06601	ASD/DD	15q11.2 dup (154 Kb)	chr15:22839023-22993121	*CYFIP1*	Strong candidate	NA	NA
CCF08207	ASD/DD	15q11.2 del (916 Kb)	chr15:22310511-23226254	*CYFIP1*	Strong candidate	15q11.2 Deletion syndrome (OMIM 615656)	HP:0001249, intellectual disability; HP:0000729, autistic behavior; HP:0001263, global DD
CCF11952	ASD/DD	15q11.1-q11.2 dup (3 Mb)	chr15:20263192-23242837	*CYFIP1*	Strong candidate	NA	NA
CCF07685	ASD/DD	2q13 del (130 Kb)	chr2:110852875-110983320	*NPHP1*	NA	PMID: 29895855	HP:0001249, intellectual disability
CCF08084	ASD/DD	2p12 dup (355 Kb)	chr2:80673046-81027675	*CTNNA2*	Syndromic	NA	HP:0001263, global DD; HP:0001321, cerebellar hypoplasia; HP:0001302, pachygyria
CCF10918	ASD/DD	8p23.1 dup (3.8 Mb)	chr8:8092788-11895164	*PINX1*	NA	8p23.1 Duplication syndrome (DECIPHER)	HP:0001263, global DD; HP:0001249, intellectual disability; HP:0000846, adrenal insufficiency
CCF05270	No ASD/DD	2q13 del (130 Kb)	chr2:110852875-110983320	*NPHP1*	NA	PMID: 29895855	HP:0001249, intellectual disability
CCF13078	No ASD/DD	15q13.3 dup (450 Kb)	chr15:32065095-32515100	*CHRNA7*	Strong candidate	15q13.3 Duplication syndrome	HP:0003829, incomplete penetrance; HP:0000256, macrocephaly; HP:0000717, autism; HP:0001263, global DD
CCF13466	No ASD/DD	1q21.1 del (356 Kb)	chr1:145383239-145738979	TAR region	NA	1q21.1 TAR deletion syndrome (OMIM 274000, DECIPHER)	HP:0006101, finger syndactyly; HP:0001263, global DD; HP:0001249, intellectual disability; HP:0007413, nevus flammeus localized in the skin of the forehead
CCF01879	No ASD/DD (cancer)	2q13 del (130 Kb)	chr2:110852875-110983320	*NPHP1*	NA	PMID: 29895855	HP:0001249, intellectual disability
		11p15.4 del (47.5 Kb)	chr11:4530773-4578227	*OR52M1*	Strong candidate	NA	NA
CCF04392	No ASD/DD (cancer)	3p21.1 del (997 Kb)	chr3:53414424-54411844	*CACNA2D3, CACNA1D*	Strong candidate	NA	HP:0001263, global DD; HP:0010864, intellectual disability, severe

Abbreviations: ASD, autism spectrum disorder; CNV, copy number variation; DD, developmental delay; DECIPHER, Database of Chromosomal Imbalance and Phenotype Using Ensembl Resources; del, deletion; dup, duplication; NA, not applicable; OMIM, Online Mendelian Inheritance in Man; PMID, PubMed identification number; TAR, thrombocytopenia-absent radius.

**Table 3.** Clinical Features of Patients With PHTS and Pathogenic and Likely-Pathogenic CNVs

Patient Identification	Germline *PTEN* Mutation^a^	Phenotype Group	Sex	Neurodevelopmental and Cognitive Features	Other Clinical Features
CCF00105	p.Lys164Argfs*3	ASD/DD	Male	Intellectual disability	Thyroid cancer (7 y of age), renal cell cancer (21 y of age), benign skin neoplasm, acral keratoses, trichilemmoma, lipoma, arteriovenous malformation, thyroid nodule
CCF00301	p.Met239Thr	ASD/DD	Male	Intellectual disability	Macrocephaly
CCF00547	Whole gene deletion	ASD/DD	Male	Global DD	Macrocephaly, dysmorphic features, epilepsy, juvenile polyps, tan macules on glans penis and penile shaft, cryptorchidism, mild chordee
CCF03028	Whole gene deletion	ASD/DD	Male	Mental retardation	Macrocephaly, dysmorphic features, juvenile polyposis syndrome, GI polyps, arteriovenous malformation, oral mucosa papilloma, tan macules on glans penis and penile shaft
CCF05681	p.Leu139Phe	ASD/DD	Male	ASD, global DD	Macrocephaly, dysmorphic features, café-au-lait spots
CCF06601	p.Ala39Thr	ASD/DD	Male	ASD, global DD	Macrocephaly, isolated hemihyperplasia, skin tag
CCF08207	p.Cys136Arg	ASD/DD	Female	Variable delay	Macrocephaly, oral mucosa papilloma, lobular breast carcinoma in situ (44 y of age), benign breast disease, goiter, ovarian cysts, skin tag
CCF11952	p.Arg130*	ASD/DD	Male	Global DD	Macrocephaly, hemangioma, lipoma
CCF07685	p.Asn12Thr	ASD/DD	Male	Global DD	Macrocephaly, dysmorphic features
CCF08084	p.Tyr68Cys	ASD/DD	Female	Global DD, anxiety disorder	Macrocephaly and dolicocephaly, café-au-lait spots, ventriculomegaly, Rathke cleft cyst, skin thickening
CCF10918	p.Thr319Asnfs*6	ASD/DD	Female	Mental retardation, global DD	Macrocephaly, dysmorphic features, hirsutism
CCF05270	p.Gly129Glu	No ASD/DD	Female	NA	Macrocephaly, GI polyps, arteriovenous malformation, benign breast disease, colon lipoma, glycogenic acanthosis, goiter, skin tag
CCF13078	p.Thr319*	No ASD/DD	Female	NA	Macrocephaly, GI polyps, benign neurologic neoplasms, benign breast disease, lipoma, neoplasms in vascular tissue, skin papilloma, thyroid nodule
CCF13466	p.Arg335*	No ASD/DD	Male	NA	Macrocephaly and dolicocephaly, Chiari malformation type I, syndactyly (toes 2 and 3), papillomatous papules, multiple melanocytic nevi, nevus flammeus of face, epidermal nevus
CCF01879	p.Arg335*	No ASD/DD (cancer)	Male	NA	Macrocephaly, thyroid cancer (69 y of age), GI polyps, angiolipoma, lipoma, basal cell carcinoma, benign neoplasm of skin, goiter, oral mucosa papilloma, skin papilloma, trichilemmoma
CCF04392	p.Arg233*	No ASD/DD (cancer)	Female	NA	Macrocephaly, thyroid cancer (42 y of age), breast cancer (44 y of age), lobular carcinoma in situ of breast, benign breast disease, benign neoplasm of skin, benign neurologic neoplasms, dermal fibroma, Hashimoto thyroiditis, lipoma

Abbreviations: ASD, autism spectrum disorder; CNV, copy number variation; DD, developmental delay; GI, gastrointestinal; NA, not applicable; PHTS, *PTEN* hamartoma tumor syndrome.

^a^ All mutations have been reported in ClinVar as pathogenic except for 2 unreported mutations (p.Met239Thr and p.Ala39Thr).

## Discussion

The present study evaluated the association of CNVs with clinical outcomes, specifically cancer vs ASD/DD, in individuals with germline pathogenic *PTEN* mutations. We found that patients with PHTS and ASD/DD have an overall increased genome-wide CNV burden per individual and an enrichment of clinically relevant CNVs when compared with the subset of patients with cancer and those without ASD/DD. These data appear to provide proof of principle that CNVs may act as genomic modifiers of disease risk in phenotypically heterogeneous hereditary disorders such as PHTS.

As many as 23% of patients with PHTS develop ASD/DD.^16^ Conversely, 2% to 5% of unselected patients with ASD/DD and as many as 50% of children with ASD and macrocephaly have been found to carry germline *PTEN* mutations.^39^ As such, *PTEN* itself is a syndromic autism gene with well-established roles in nervous system development and neuronal function.^40^ However, why more than 75% of *PTEN* mutation carriers do not develop ASD/DD remains a clinical conundrum. Our findings suggest pathogenic and likely pathogenic CNVs are associated with ASD/DD risk regardless of the particular coexisting germline *PTEN* mutation. Autism spectrum disorder is believed to be a highly heritable disorder, with complex genetic and biological bases.^41,42,43^ Pathogenic CNVs have been identified in 8% to 21% of individuals with idiopathic ASD, with higher frequencies reported in more severe cases, including causing syndromic ASD.^9,44,45^ Notably, particular pathogenic CNVs have also been identified in neurotypical control individuals, including unaffected family members, albeit with significantly lower frequencies.^46^ First, this observation corroborates the incomplete penetrance and extreme variability in expression of the associated CNVs, meaning that not all carriers will manifest the associated clinical features. Second, this observation supports the burgeoning hypothesis that ASD etiology and severity are modulated through additive effects of multiple genomic aberrations, the so-called 2-hit (or multiple-hit) model.^47,48,49^ These characteristics are highly relevant in the context of modifiers within an inherently predisposed genetic background. In the case of PHTS, we found evidence for the association of pathogenic CNVs with the coexisting germline *PTEN* mutations to favor ASD/DD clinical outcomes.

For example, we identified CNVs involving *CYFIP1* (OMIM 606322) in 3 unrelated patients with PHTS in the ASD/DD group. *CYFIP1* encodes cytoplasmic FMR1-interacting protein 1, a protein originally found to interact with fragile X mental retardation protein.^50^ Increased *CYFIP1* dosage has been shown to cause aberrant neuronal cellular and dendritic morphologic features and to be associated with ASD in humans and murine model organisms.^51,52^ Importantly, *CYFIP1* expression was inversely correlated with *PTEN* expression levels, with concomitant dysregulation of mammalian target of rapamycin (mTOR) signaling,^51^ suggesting that such CNVs may represent a second hit for patients with PHTS already harboring deleterious *PTEN* mutations that upregulate AKT-mTOR signaling. In cases of *CYFIP1* duplications, decreased PTEN protein levels could also affect wild-type *PTEN*. In the context of *CYFIP1* deletion, as was observed for 1 of the 3 patients with PHTS and CNVs involving *CYFIP1* in this study, increased levels of a mutant PTEN protein may result in dominant negative effects,^53^ inhibiting wild-type *PTEN* and hence exacerbating the condition.

Notwithstanding the association of CNVs with multiple neurodevelopmental disorders, CNVs have also been implicated in the etiology of neuropsychiatric diseases such as epilepsy, schizophrenia, and bipolar disorder.^11,29,54,55^ A broad spectrum of neuropsychiatric phenotypes has been observed in patients with PHTS ranging from normal development and intelligence to severely debilitating neurocognitive and motor aberrations, including generalized anxiety, adult-onset movement disorders, obsessive-compulsive disorder, and psychosis.^56,57^ Whether particular CNVs affect the spectrum or severity of such motor and/or neurocognitive disease manifestations in PHTS warrants further scrutiny in larger independent prospective series.

### Limitations

One limitation of this study is the relatively small sample size of patients within each phenotype group, owing to the rarity of PHTS, despite our cohort being the largest globally to our knowledge. Cowden syndrome, the most common component disorder in PHTS, is estimated to have a prevalence of 1 per 200 000 individuals.^58^ This precludes us from performing in-depth analyses, such as the association of particular CNVs with specific phenotypes or severity of manifestations within the ASD/DD group. Another limitation is the absence of a large independent replication sample to ascertain our findings. We therefore attempted a minivalidation by analyzing 69 genetically determined non-European patients with PHTS excluded from the above presented data to control for population stratification (eTable 3 in the Supplement). We similarly found that 3 of 28 patients in the ASD/DD group (10.7%) carried pathogenic and/or likely pathogenic CNVs compared with 1 of 41 patients in the no ASD/DD group (2.4%) (eTable 4 in the Supplement). The clinical relevance of increased duplications in genic regions in the ASD/DD group compared with the no ASD/DD group remains unanswered. Other loci may be implicated in the etiology of ASD/developmental delay in PHTS, and therefore the extent of CNVs in our study is likely an underestimate through the conservative inclusion of only pathogenic and/or likely pathogenic CNVs in this study.

## Conclusions

The identification of testable clinically relevant genomic modifiers in PHTS is important for risk stratification and serves as proof of principle for other phenotypically variable disorders. Patients with PHTS and ASD/DD have an increased burden of pathogenic and/or likely pathogenic CNVs associated with neurodevelopmental disorders. Although CNV analysis is indicated as a first-tier clinical diagnostic test for patients with unexplained ASD/DD,^13^ it is not standard to test for such genomic alterations in the setting of an established diagnosis with a syndromic high-penetrance mendelian gene such as *PTEN*. Copy number variation analysis on immediate identification of germline *PTEN* mutation in infants and children may be beneficial. The presence of pathogenic and/or likely pathogenic CNVs in PHTS would suggest high likelihood of ASD/neurodevelopmental delay, leading to timely referral to formal evaluation and, if the results are positive, subsequent therapy. The earlier such interventions are instituted, the better the outcomes.

## Notes

Supplement. **eFigure 1.** Workflow for CNV Discovery, Quality Control, and Downstream Analyses **eFigure 2.** *PTEN* Mutation Spectra Across the 3 PHTS Clinical Phenotype Groups **eTable 1.** Demographic and Clinical Characteristics of 481 PHTS Patients **eTable 2.** Patients With PHTS and Germline Pathogenic and Likely Pathogenic CNVs in Cancer-Predisposing Genes **eTable 3.** Demographic and Clinical Characteristics of 69 Patients With PHTS of Non-European Ancestry **eTable 4.** Pathogenic and Likely Pathogenic CNVs Identified in Patients With PHTS of Non-European Ancestry
