# Supplementary material for: Copy Number Variation and Clinical Outcomes in Patients With Germline PTEN Mutations
Source: JAMA Netw Open. 2020 Jan 31;3(1):e1920415. doi: 10.1001/jamanetworkopen.2019.20415 (PMC7042875; doi:10.1001/jamanetworkopen.2019.20415)
Supplement: Supplement. — eFigure 1. Workflow for CNV Discovery, Quality Control, and Downstream Analyses eFigure 2. PTEN Mutation Spectra Across the 3 PHTS Clinical Phenotype Groups eTable 1. Demographic and Clinical Characteristics of 481 PHTS Patients eTable 2. Patients With PHTS and Germline Pathogenic and Likely Pathogenic CNVs in Cancer-Predisposing Genes eTable 3. Demographic and Clinical Characteristics of 69 Patients With PHTS of Non-European Ancestry eTable 4. Pathogenic and Likely Pathogenic CNVs Identified in Patients With PHTS of Non-European Ancestry [file jamanetwopen-3-e1920415-s001.pdf]

Supplementary Online Content

Yehia L, Seyfi M, Niestroj L-M, et al. Copy number variation and clinical outcomes in patients with germline *PTEN* mutations. *JAMA Netw Open*. 2020;3(1):e1920415.

doi:10.1001/jamanetworkopen.2019.20415

eFigure 1. Workflow for CNV Discovery, Quality Control, and Downstream Analyses

eFigure 2. *PTEN* Mutation Spectra Across the 3 PHTS Clinical Phenotype Groups

eTable 1. Demographic and Clinical Characteristics of 481 PHTS Patients

eTable 2. Patients With PHTS and Germline Pathogenic and Likely Pathogenic CNVs in Cancer-Predisposing Genes

eTable 3. Demographic and Clinical Characteristics of 69 Patients With PHTS of Non-European Ancestry

eTable 4. Pathogenic and Likely Pathogenic CNVs Identified in Patients With PHTS of Non-European Ancestry

This supplementary material has been provided by the authors to give readers additional information about their work.

© 2020 Yehia L et al. *JAMA Network Open*.

eFigure 1. Workflow for CNV Discovery, Quality Control, and Downstream Analyses

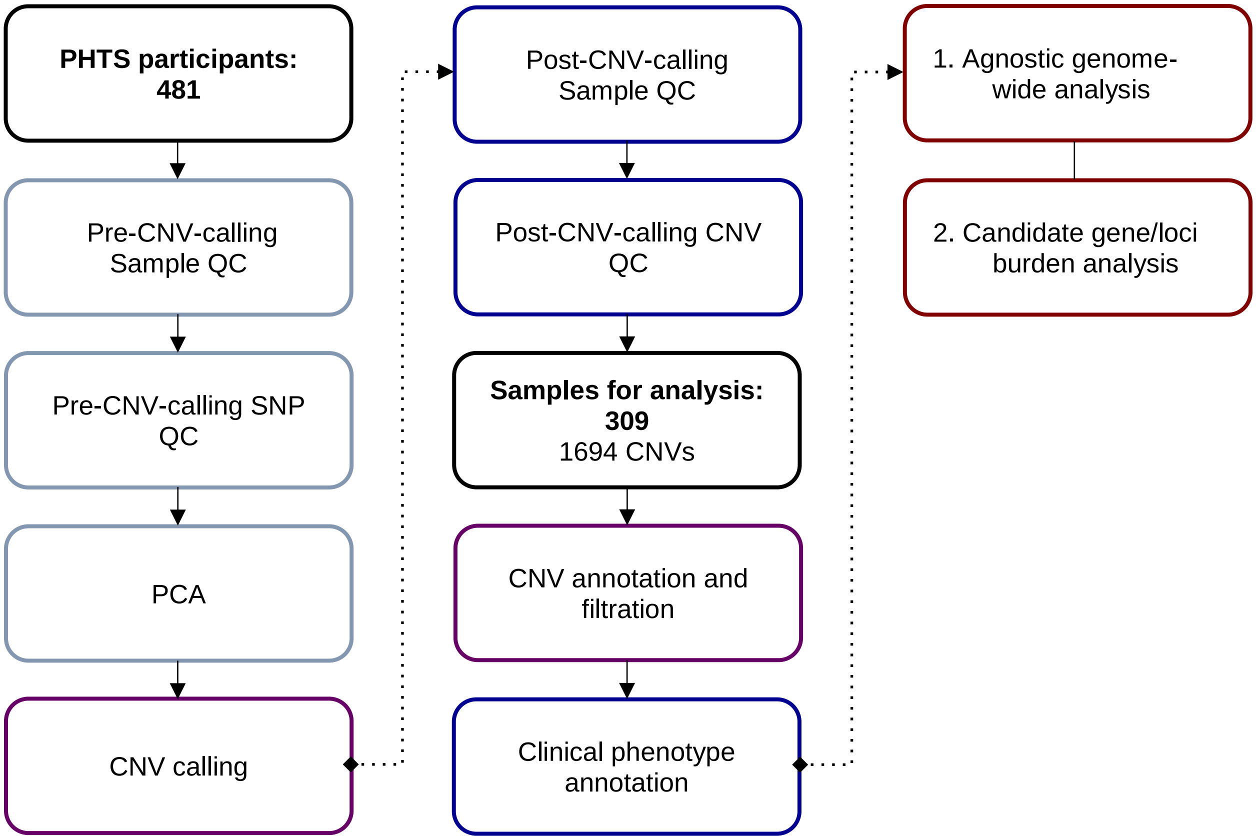

Abbreviations: PHTS, *PTEN* hamartoma tumor syndrome; CNV, copy number variation; QC, quality control; SNP, single nucleotide polymorphism; PCA, principal component analysis.

© 2020 Yehia L et al. *JAMA Network Open*.

eFigure 2. *PTEN* Mutation Spectra Across the 3 PHTS Clinical Phenotype Groups

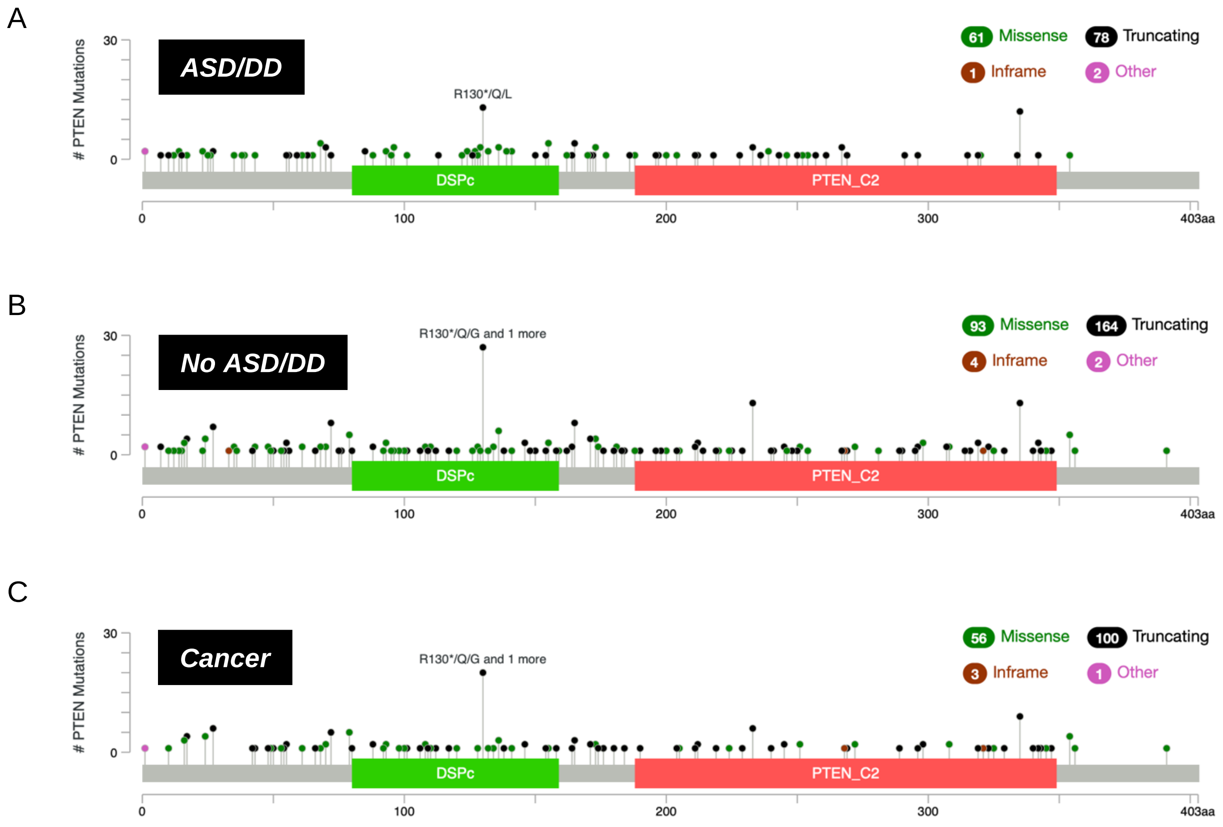

PTEN mutations are depicted in the lollipop plots overlying the PTEN protein structures. The frequency of each mutation correlates with the heights of the vertical lines representing each lollipop. A, *PTEN* mutations identified in PHTS individuals with ASD/DD. Three whole *PTEN* gene deletions, one exon 1 deletion, and one exon 1 duplication are not depicted. B, *PTEN* mutations identified in PHTS individuals without ASD/DD, including the subset with cancer. Two whole *PTEN* gene deletions, three exon 1 deletions, two exon 1-2 deletions, one exon 6 duplication, one exon 2-9 deletion, and 21 promoter mutations are not depicted. C, *PTEN* mutations identified in PHTS individuals with cancer. One whole *PTEN* gene deletion, one exon 1 deletion, one exon 1-2 deletions, one exon 6 duplication, and 20 promoter mutations are not depicted. We utilized the cBioPortal MutationMapper tool to depict the identified *PTEN* germline mutations (https://www.cbioportal.org/mutation_mapper). Family members were excluded from each PHTS phenotype group. The ‘other’ category includes the *PTEN* p.M1I start loss mutation.

Abbreviations: ASD, autism spectrum disorder; DD, developmental delay.

© 2020 Yehia L et al. *JAMA Network Open*.

eTable 1. Demographic and Clinical Characteristics of 481 PHTS Patients

Clinical phenotypic characteristics	Number (%)
Gender	
Female	268 (55.7%)
Male	213 (44.3%)
Mean age at consent (SD, range)	33 (22, 1-85)
Mean CC score (SD, range)	20 (13, 0-69)
Neurodevelopmental features	157 (32.6%)
Autism spectrum disorder	57
Global developmental delay	76
Variable delay	29
Mental retardation	13
Learning disabilities	9
Cancer[a]	207 (43.0%)
PHTS component malignancies	
Breast cancer	90
Thyroid cancer	60
Renal cell cancer	29
Endometrial cancer	35
Colon cancer	17
Melanoma	10
Non-malignant features[b]	132 (27.4%)
Macrocephaly	102
Dermatologic features[c]	82
Arteriovenous malformations	15
Hemangiomas	25
Polyposis	59
Benign breast features[d]	27
Benign thyroid features[e]	88
Lhermitte-Duclos disease	14

[a]*Includes fifteen patients with neurodevelopmental disorders who have also been diagnosed with cancer.*
[b]*Patients without a personal history of neurodevelopmental disorders or cancer at the time of the last clinical visit and/or follow-up.*
[c]*Includes trichilemmoma, acral keratosis, papillomatous papules, and genital lentiginosis (penile freckling in males).*
[d]*Includes breast fibroadenoma, fibrocystic breast disease, breast papilloma, breast hamartoma, atypical ductal hyperplasia, and typical ductal hyperplasia.*
[e]*Includes thyroid nodules, goiter, and Hashimoto thyroiditis.*

Abbreviations: PHTS, *PTEN* hamartoma tumor syndrome; SD, standard deviation; CC score, Cleveland Clinic score (Tan MH, Mester J, Peterson C, et al. A clinical scoring system for selection of patients for *PTEN* mutation testing is proposed on the basis of a prospective study of 3042 probands. *Am J Hum Genet.* 2011;88(1):42-56).

© 2020 Yehia L et al. *JAMA Network Open*.

eTable 2. Patients With PHTS and Germline Pathogenic and Likely Pathogenic CNVs in Cancer-Predisposing Genes

Patient ID	Sex	Age[a]	Clinical Phenotypes	CNV Characteristics
CCF00547-01-001	M	3	Macrocephaly, dysmorphic features, global developmental delay, tan macules on glans penis and penile shaft, juvenile polyps, congenital genitourinary tract anomalies, epilepsy	Gene: ***BMPR1A*** (OMIM 601299)<br><br>chr10:85624500-90412968<br>Length: 4.8 Mb<br>Type: Deletion
CCF03028-01-001	M	21	Macrocephaly, dysmorphic features, mental retardation, tan macules on glans penis and penile shaft, oral mucosa papilloma, adenomatous polyps, colon ganglioneuroma, arteriovenous malformation, inflammatory polyp, juvenile polyp, hyperplastic benign polyp, juvenile polyposis syndrome	Gene: ***BMPR1A*** (OMIM 601299)<br><br>chr10:87976544-91250370<br>Length: 3.3 Mb<br>Type: Deletion
CCF01852-01-001	F	6	Macrocephaly, dysmorphic features, global developmental delay, connective tissue nevus (NOS), lipoma	Gene: ***BRCA1*** (OMIM 113705)<br><br>chr17:41256153-41319650<br>Length: 63.5 Kb<br>Type: Deletion

[a]Age refers to the age at consent in years.

Abbreviations: CNV, copy number variation; NOS, not otherwise specified; OMIM, Online Mendelian Inheritance in Man; chr, chromosome.

© 2020 Yehia L et al. *JAMA Network Open*.

eTable 3. Demographic and Clinical Characteristics of the Analytical Series of 69 Patients With PHTS of Non-European Ancestry

Clinical phenotypic characteristics	Number (%)
Gender	
Female	33 (47.8%)
Male	36 (52.2%)
Mean age at consent (SD, range)	26 (19, 1-72)
Germline PTEN mutations	
Promoter	0
Missense	20
Nonsense	18
Splice site	7
Frameshift truncating	18
Indels	6
Neurodevelopmental features	28 (40.6%)
Autism spectrum disorder	7
Global developmental delay	14
Variable delay	7
Mental retardation	5
Learning disabilities	3
Cancer[a]	19 (27.5%)
PHTS component malignancies	
Breast cancer	6
Thyroid cancer	8
Renal cell cancer	0
Endometrial cancer	2
Colon cancer	4
Melanoma	0
Non-malignant features[b]	24 (34.8%)
Macrocephaly	20
Dermatologic features[c]	10
Arteriovenous malformations	4
Hemangiomas	7
Polyposis	12
Benign breast features[d]	3
Benign thyroid features[e]	11
Lhermitte-Duclos disease	3

[a] *Includes two patients with neurodevelopmental disorders who have also been diagnosed with cancer.*
[b] *Patients without a personal history of neurodevelopmental disorders or cancer at the time of the last clinical visit and/or follow-up.*
[c] *Includes trichilemmoma, acral keratosis, papillomatous papules, and genital lentiginosis (penile freckling in males).*
[d] *Includes breast fibroadenoma, fibrocystic breast disease, breast papilloma, breast hamartoma, atypical ductal hyperplasia, and typical ductal hyperplasia.*
[e] *Includes thyroid nodules, goiter, and Hashimoto thyroiditis.*

Abbreviations: PHTS, *PTEN* hamartoma tumor syndrome; SD, standard deviation; Indels, insertions and deletions.

© 2020 Yehia L et al. *JAMA Network Open*.

eTable 4. Pathogenic and Likely Pathogenic CNVs Identified in Patients With PHTS of Non-European Ancestry

Patients[a]	Clinical Features	CNV (size)	Genomic Coordinates (hg19)	Associated Genes	Associated Genomic Syndromes	ASD/DD-Related Human Phenotype Ontology (HPO)
CCF06052-01-001<br>2 yo. M<br>ASD/DD	Macrocephaly, global developmental delay, hamartomatous polyp, skin tag, tan macules on glans penis and penile shaft	15q13.3 dup (490 Kb)	chr15:32025034-32515100	*CHRNA7*	15q13.3 duplication syndrome	HP:0003829, Incomplete penetrance; HP:0000256, Macrocephaly; HP:0000717, Autism; HP:0001263, Global developmental delay
CCF00068-01-001<br>54 yo. F<br>No ASD/DD (cancer)	Breast cancer (age 50), goiter, endometrial polyp, Lhermitte-Duclos disease, multiple esophageal plaques, papilloma	22q11.21 del (127 Kb)	chr22:18875445-19002159	*PRODH*	NA	HP:0001249, Intellectual disability; HP:0000729, Autistic behavior; HP:0001263, Global developmental delay
CCF07579-01-001<br>8 yo. F<br>ASD/DD	Macrocephaly, ASD, variable delay, learning disabilities, goiter, acral keratoses, benign neoplasm of skin, GI polyps, skin tag, hemangioma of skin, hyperpigmentation, juvenile polyposis, supernumerary permanent teeth	10q23.2 del (4.7 Mb)	chr10:86195513-90920948	*GLUD1/ GRID1*	10q23.2 deletion syndrome	HP:0000256, Macrocephaly; HP:0000717, Autism; HP:0001263, Global developmental delay; HP:0001249, Intellectual disability; HP:0200008, Intestinal polyposis
CCF04850-01-001<br>2 yo. M<br>ASD/DD	Macrocephaly, variable delay, epilepsy, lipoma, hyperpigmentation, tan macules on glans penis and penile shaft, hypospadias, unilateral hypotrophic kidney	20p11.21 del (155 Kb)	chr20:25451067-25606369	*NINL*	NA	NA

[a]Ages in years correspond to the age at consent for each patient.

Abbreviations: CNV, copy number variation; yo., years old; ASD, autism spectrum disorder; DD, developmental delay; chr, chromosome; NA, not applicable.

© 2020 Yehia L et al. *JAMA Network Open*.
